# Emerging roles of fibroblasts in cardiovascular calcification

**DOI:** 10.1111/jcmm.16150

**Published:** 2020-12-23

**Authors:** Wudi Li, Sheng‐an Su, Jian Chen, Hong Ma, Meixiang Xiang

**Affiliations:** ^1^ Department of Cardiology The Second Affiliated Hospital Zhejiang University School of Medicine Hangzhou China

**Keywords:** calcification, fibroblasts, heart, valve, vasculature

## Abstract

Cardiovascular calcification, a kind of ectopic mineralization in cardiovascular system, including atherosclerotic calcification, arterial medial calcification, valve calcification and the gradually recognized heart muscle calcification, is a complex pathophysiological process correlated with poor prognosis. Although several cell types such as smooth muscle cells have been proven critical in vascular calcification, the aetiology of cardiovascular calcification remains to be clarified due to the diversity of cellular origin. Fibroblasts, which possess remarkable phenotypic plasticity that allows rapid adaption to fluctuating environment cues, have been demonstrated to play important roles in calcification of vasculature, valve and heart though our knowledge of the mechanisms controlling fibroblast phenotypic switching in the calcified process is far from complete. Indeed, the lack of definitive fibroblast lineage‐tracing studies and typical expression markers of fibroblasts raise major concerns regarding the contributions of fibroblasts during all the stages of cardiovascular calcification. The goal of this review was to rigorously summarize the current knowledge regarding possible phenotypes exhibited by fibroblasts within calcified cardiovascular system and evaluate the potential therapeutic targets that may control the phenotypic transition of fibroblasts in cardiovascular calcification.

## 1 INTRODUCTION

Cardiovascular disease (CVD) is the leading cause of mortality in the world, which has brought immense burdens on global public health.^1^ With the increase of ageing population, the incidence of CVD is dramatically rising. In 2015, there were ~400 million CVD cases and ~18 million CVD‐related deaths.^2^ The calcification of cardiovascular system is an important risk factor for adverse cardiovascular events. Severe calcification in cardiovascular system predicts a poor prognosis in CVD patients.^3^ Therefore, great efforts are being made to dissect the molecular mechanism underlying cardiovascular calcification and to improve CVD prognosis via harnessing or even reversing the ectopic calcification.

Like bone formation, ectopic calcification, including cardiovascular calcification, is also a complex regulated process of ossification characterized by calcification of extracellular matrix (ECM). It is a tightly regulated, active process mediated by cells that are capable of mineralization in soft tissues. Mesenchymal cells are the major sources of ectopic calcification, such as vascular smooth muscle cells (VSMCs), valve interstitial cells (VICs) and dermal fibroblasts. All these cells can transit into osteoblast‐like cells with up‐regulation of osteogenic markers.^4^, ^5^, ^6^ However, the situation is more complicated in cardiovascular calcification based on its cellular diversity.

The most common forms of cardiovascular calcification include vascular calcification, valvular calcification and myocardial calcification. Vascular calcification, with the deposition of hydroxyapatite in arterial wall, can be classified into two completely different forms: intimal calcification and medial calcification, depending on the location of calcified minerals. Valvular calcification mainly affects aortic and mitral valves, which refers to calcific aortic valve disease (CAVD) and mitral annular calcification (MAC). Last, but not the least, myocardial calcification is another form of cardiovascular calcification. It is being recognized as a novel adverse sign of ventricular remodelling. Myocardial calcification, mostly accompanied by fibrosis, can interrupt the electrical propagation of the conduction system. It leads to arrhythmia and cardiac dysfunction.^7^ Therefore, cardiovascular calcification is a serious condition affecting major components of cardiovascular system. However, the aetiology of cardiovascular calcification is still unclarified.

## 2 THE MAIN SOURCES OF CALCIFICATION

Ectopic calcification, which refers to the mineralization of extracellular matrix (ECM) occurred in areas other than skeleton and teeth, is also named as soft‐tissue calcification. It could be classified as (a) metastatic calcification, which arises in accompany with up‐regulated serum calcium or phosphate; (b) dystrophic calcification, which mainly exists in chronically damaged tissues with normal serum calcium/phosphate levels; and (c) tumoral calcinosis (TC), an uncommon clinicopathological condition, which is characterized by the deposition of calcified masses in juxta‐articular and a normal calcium/phosphate level in serum.^8^, ^9^

Smooth muscle cells and fibroblasts are the main players in ectopic calcification. They mediate the abnormal mineral deposition through a common process, which is an imbalance between pro‐calcification stimuli and calcification inhibitors. Hydroxyapatite is the main crystal phase presented in ectopic mineralized ECM, which is predominantly constituted by calcium (Ca) and inorganic phosphate (Pi) ions. Pi plays a critical role in triggering ectopic ECM mineralization. However, simply elevating the concentration of phosphate is insufficient to induce ECM mineralization because of the existence of inorganic pyrophosphate (PPi). PPi is a strong inhibitor of ectopic mineralization which could directly inhibit the aggregation of hydroxyapatite and crystal growth.^10^, ^11^ Thus, the dynamic alterations of Pi/PPi ratio is critical in regulating the ectopic mineralization process. Most anti‐mineralization and pro‐mineralization factors function by altering the ratio of Pi/PPi, such as alkaline phosphatase (ALP), matrix Gla protein (MGP), ectonucleotide pyrophosphatase/phosphodiesterase‐1 (ENPP1) and pyrophosphate analogues.^12^, ^13^, ^14^, ^15^ ALP promotes matrix mineralization by degrading inorganic pyrophosphate and producing mineral constituents of hydroxyapatite‐Pi. ENPP1 inhibits ectopic mineralization by increasing PPi supplementation. Bisphosphonate can be used as an anti‐mineralization agent by mimicking PPi.^15^ On the contrary, in skeleton and teeth, PPi promotes mineralization cause it can be broken down by tissue non‐specific alkaline phosphatase (TNAP) to generate Pi.^14^ There still exist other factors that can inhibit matrix mineralization without directly interfering the ratio of Pi/PPi, for instance fetuin‐A. Fetuin‐A inhibits de novo formation of hydroxyapatite crystals by forming transient‐soluble, colloidal spheres (containing Ahsg, calcium, phosphate) and delaying their precipitation.^16^, ^17^ Osteopontin inhibits mineralization through directly binding to crystal surfaces and inhibiting any further propagation.^13^, ^18^ These pathways provide a strong possibility to better understand the calcification process.

### 2.1 Roles of vascular smooth muscle cells in vascular calcification

Vascular calcification, with the deposition of hydroxyapatite in arterial wall, can be classified into two completely different forms depending on the location of minerals, including intimal calcification and medial calcification. Vascular calcification is strongly associated with acute cardiovascular and cerebrovascular events, especially combined with diabetes and chronic kidney disease.^19^, ^20^ It is closely correlated with increased all‐cause and cardiovascular morbidity and mortality.^21^

The walls of large and moderate arteries have three distinct layers: the tunica intima, tunica media and tunica adventitia. The intimal layer is majorly composed of endothelial cells. The media, also named the smooth muscle layer, is composed of concentric rings of smooth muscle cells and collagen, elastin fibres. The adventitia, the most complicated layer of vessel walls, is composed of fibroblasts, vascular progenitor cells and immune cells.^22^, ^23^ Intimal calcification is associated with the forming of atherosclerotic plaque. It frequently occurs in the coronary and carotid arteries, exacerbating arterial stenosis or obstruction. Coronary artery calcification is an important part of intimal calcification, which can be evaluated by overall coronary artery calcium score using non‐invasive imaging. Coronary calcium score is a critical index to identify high‐risk atherosclerotic CVD patients.^24^, ^25^ Medial calcification can lead to vessel stiffening and decreased compliance, occurring in extremity arteries such as tibial and femoral, causing insufficient blood supply.

Osteogenic differentiation of VSMCs is likely a common feature of intimal and medial calcification. However, stimuli that induce this process are different, and intimal calcification is often induced by disrupted lipid homeostasis, oxidative stress and inflammation.^26^ Medial calcification is usually accompanied by ageing, diabetes and renal disease.^27^ For vasculature, it has gained acceptance that VSMCs undergo ‘de‐differentiation into a synthetic phenotype and further step into osteogenic fate’ during vascular calcification. The phenotypic transition of VSMCs is accompanied by the increased expression of bone markers, such as ALP, collagen 1, runt‐related transcription factor‐2 (Runx2), osteopontin, osteocalcin and reduced expression of VSMCs markers including SM22α, smooth muscle α‐actin and smooth muscle cell myosin heavy chain.^28^ The identical roles of VSMCs in intimal and medial calcification has been illustrated by others.^29^ Many factors can inhibit vascular calcification by re‐balancing calcium‐phosphate metabolism or inhibiting the formation and propagation of hydroxyapatite crystals, such as MGP, osteopontin, fetuin‐A, ENPP1 and pyrophosphate analogues.^12^, ^13^, ^14^, ^15^, ^17^

### 2.2 Roles of fibroblasts in ectopic calcification

Ectopic soft‐tissue calcification is also a characteristic sign of many diseases, such as Werner's syndrome (WS) and pseudoxanthoma elasticum (PXE). Werner's syndrome, a rare autosomal recessive disorder, caused by mutations in the *WRN* gene, is often accompanied by extensive subcutaneous calcification,^30^ whereas pseudoxanthoma elasticum is caused by the *ABCC6* gene mutation and characterized as progressive calcification of elastic fibres in skin, eyes and the cardiovascular system.^31^ Besides, gamma‐glutamyl carboxylase (GGCX) syndrome, caused by *GGCX* gene mutation, also possess PXE‐like symptoms, with calcium deposits in vessel walls and elastic fibres.^32^ In the occurrence of dermal ectopic calcification, fibroblasts are concerned as principal candidates.^33^ Dermal fibroblasts treated with pro‐calcifying medium for 3 weeks exhibited evident calcium deposits.^34^, ^35^ Interestingly, Federica et al found that dermal fibroblasts derived from PXE patients are more susceptible to mineralization stimuli.^36^ Yumi et al indicated that dermal fibroblasts derived from GGCX patients have higher ALP activity and mRNA levels of many osteogenic markers under osteogenic induction, showing elevated sensitivity to calcific stimulus. Meanwhile, the cultured WS fibroblasts were reported to exhibit spontaneous mineralization in vitro under a normal phosphate concentration by Satoshi et al, suggesting that these fibroblasts cultured in vitro retain several pathologic characteristics which they have in vivo.^33^, ^37^

Besides, liver calcification following human liver transplantation may lead to graft dysfunction or even graft failure. Fariba et al reported that myofibroblasts were over‐agglomerated in pre‐calcified and calcified areas with large expression of bone‐specific matrix proteins and transcription factors, suggesting that liver calcification after graft may be the consequence of a generalized response to stimuli involving osteogenic differentiation of activated myofibroblasts.^38^, ^39^, ^40^ Meanwhile, fibroblasts derived from spinal ligaments also possess a strong mineralizing potential. Mechanical stress induces the osteogenic differentiation of ligament fibroblasts through activating Cx43/MAPK/ERK signalling, knock‐down of Cx43 significantly blocked this phenomena. IL‐6 can also promote the ossification of ligament fibroblast by activating MAPK/ERK pathway, which is the major component of osteogenic signalling pathways, demonstrating that the osteoblastic differentiation of fibroblasts is similar in different organs.^41^, ^42^, ^43^

## 3 EMERGING ROLES OF FIBROBLASTS IN VASCULAR CALCIFICATION

Vascular calcification is practically in parallel with the progression of atherosclerosis. Calcified lesions in atherosclerotic plaques most likely appear at the late stage of atherosclerosis. According to the diameter of calcified particles, calcified lesions are divided into macroscopic calcification and microcalcification. Macroscopic calcification, which is defined as calcified particles >50 µm in diameter,^44^ makes plaque more stable. μCalcs (microcalcification) seems to be harmful when it is embedded in the fibrous cap, which can increase the vulnerability of plaque by increasing local stress over 2‐5 times.^45^, ^46^ Once the local stress grows to over 300 kPa, plaque rupture may happen.^47^

The roles of intima and media in atherosclerosis have attracted great attention, whereas the emerging roles of adventitia in atherosclerosis are being clarified. Li et al demonstrated that local overexpression of adiponectin gene in adventitia via the approach of adenovirus (Ad‐APN) could dramatically reduce atherosclerotic plaque areas in rabbit models of abdominal aorta balloon injury, implying a critical role of adventitia in atherosclerosis.^48^ As the main cells in adventitia, adventitial fibroblasts (AFs) play an important role in the pathological progression of atherosclerosis. In response to many cytokines and growth factors (ie ROS, transforming growth factor ‐beta1 (TGF‐β1), tumour necrosis factor‐α (TNF‐α), fibroblast growth factor‐2 (FGF‐2), osteocalcin),^49^, ^50^, ^51^, ^52^ AFs can trans‐differentiate into myofibroblasts and migrate towards the lumen, contributing to the neointima formation in injury‐induced and graft‐induced atherosclerosis.^53^ Besides, Karamariti et al revealed that the transplanted AFs can migrate to the intima and trans‐differentiate into smooth muscle cells in mouse wire–induced femoral artery injury model.^54^ In atherosclerotic lesions, fibroblasts can be derived from endothelial cells through endothelial‐to‐mesenchymal transition (EndMT). By using lineage‐tracking system, Evrard et al revealed that EndMT is common in atherosclerotic lesions. Higher incidence of EndMT transition is closely related to a higher risk of plaque rupture in human.^55^

The vasa vasorum (VV) provides nutrients and oxygen to adventitia and the outer part of media of large arteries, and removes waste products. AFs might play a critical role in regulating the expansion of vasa vasorum in atherosclerotic disease. The potential mechanism might be associated with AF‐secreted pro‐inflammatory cytokines and pro‐angiogenic growth factors, including interleukin‐6 (IL‐6) and vascular endothelial growth factor (VEGF).^56^, ^57^ Li et al reported that AF‐derived VEGF plays a pivotal role in the increase of VV count.^58^ Studies have discovered that the neovessels of VV contribute to plaque progression by delivering inflammatory cells to the plaques, and the leakage of immature VV is responsible for intraplaque haemorrhage. A recent study suggested that lysed erythrocytes promoted the osteoblastic differentiation of VSMCs in a NO‐dependent manner.^59^, ^60^ Furthermore, the early calcified human atherosclerotic plaques contained large amounts of platelets due to plaque neovascularization by leaky vessels, blood extravasation and haemorrhage. In patients with carotid artery stenosis, levels of osteocalcin content in circulating platelets and total osteocalcin release after activation were significantly increased. Furthermore, the overexpression of osteocalcin dramatically accelerated the mineralization of vascular smooth muscle cells (MOVAS).^61^, ^62^ Besides, by using scanning electron microscope (SEM) and immunogold, researchers found that plenty of activated platelets adhere to the surface of mineralized aortic valves and secrete numerous atherogenic mediators, which potently promote the mineralization of VIC by releasing soluble factors. Specifically, intravenous injections of activated platelets significantly promote the progression of calcific aortic valve stenosis in mice.^63^ Blocking angiogenesis of VV using angiostatin can alleviate the progression of atherosclerosis.^64^

Thus, adventitial fibroblasts, contributing to the progression of atherosclerosis, may potentially participate in vascular calcification. Haurani et al proved that AF‐derived NADPH and reactive oxygen species (ROS) are key sensors and messengers for initial remodelling of vascular disease.^65^, ^66^ Hydrogen peroxide (H_2_O_2_), a cell‐permeable ROS, which has been proved to induce VSMCs calcification by increasing the expression and activity of Runx2, a key osteogenic transcription factor controlling the expression of many osteoblastic differentiation‐related genes.^67^ ROS can also trigger the inflammatory response of adventitia via increasing the expression of interleukin‐6 (IL‐6), TNF‐α and vascular cell adhesion molecule‐1 (VCAM‐1).^68^ TNF‐α has been demonstrated to promote arterial calcification in type II diabetes (T2DM) mouse model through enhancing Msx2‐Wnt signalling.^69^ Meanwhile, IL‐6 can induce the trans‐differentiation of VSMCs into osteoblast through STAT3/JMJD2B pathway.^70^

## 4 FIBROBLASTS IN VALVE CALCIFICATION

Valve calcification is a common cardiac disease attributed by valve cells, leading to the thickness, calcification and mechanical dysfunction of valve leaflets.^71^ Calcific aortic valve disease (CAVD) is the most common form of valvular calcification with calcific nodules depositing on the cusps of the leaflets, resulting in thickened and stiffened leaflets, a remarkable reduction in valve function and ultimate aortic stenosis. Mitral annular calcification (MAC) is a relatively moderate process which occurs on the fibrous base of the mitral valve and shows rare effects on mitral valve function.^72^

Interestingly, both CAVD and MAC are tightly associated with great atherosclerotic risk factors such as hypertension, hyperlipidaemia and diabetes.^73^, ^74^, ^75^ Furthermore, MAC shares plenty of commonalities with atherosclerosis. Both are triggered by endothelium injury and result in lipid accumulation and calcium deposition. Hence, it is commonly believed that MAC is another form of atherosclerosis.^76^, ^77^ MAC is also discovered to increase the incidence of arrhythmias in similar to myocardial calcification.^78^ In patients with severe myocardial infarction, mitral valve thickness is always witnessed, leading to mitral valve regurgitation, a severe complication.^79^ Besides, multiethnic cohort studies revealed that MAC is robustly associated with CVD.^80^, ^81^ The initiating process of CAVD involves leaflets’ fibrotic changes, and granules of mineralization occur mainly in the fibrotic region of leaflets.^82^

According to the diverse morphologies, valve cells can be divided into several cell populations. Valve interstitial cell is recognized as the major cellular origin of calcific valve, which is capable of undergoing osteogenic differentiation in response to the microenvironmental and mechanical cues.^83^ However, recent study showed that valve‐derived stromal cells (VDSCs), as uniform spindle‐shaped fibroblasts, exhibiting potent proliferating ability and expressing both mesenchymal and osteogenic markers, may potently contribute to valve calcification.^84^ In addition, valve ECs also showed the potential of osteogenic differentiation via EndMT process.^85^, ^86^

## 5 FIBROBLASTS IN MYOCARDIAL CALCIFICATION

Pathological cardiac calcification is often observed in the aged and in patients with end‐stage renal disease who have disturbed calcium‐phosphate metabolism or for unknown reasons.^87^, ^88^ With calcium deposited in the myocardium, heart can be the only organ with significant calcium deposition. The deposition of calcium in myocardium is often associated with poor outcome, such as arrhythmia or sudden cardiac death.^87^ Cardiac calcification has been proved as a predictor of poor outcome following myocarditis or myocardial infarction.^87^, ^89^ However, in myocardial calcification, few is known regarding the identification of cellular source.^90^

Heart is a highly organized structure that contains several types of cells. Adult mammalian heart is mainly composed of cardiomyocytes, fibroblasts, endothelial cells (ECs) and leucocytes. Fibroblasts constitute 20‐30% of total non‐cardiomyocytes in the heart.^91^ They form the cardiac scaffold and play a critical role in heart development by producing extracellular matrix and expressing various cytokines.^92^

In response to pressure overload or ischaemic injury, the heart undergoes a dynamic remodelling process, producing a multitude of ECM proteins, such as collagens and fibronectin. The deposition of ECM is mainly driven by cardiac fibroblasts. Under stress, cardiac fibroblasts transform into myofibroblasts with elevated production of ECM and contractility, resulting in cardiac fibrosis.^93^, ^94^ Ectopic mineralization is characterized by hydroxyapatite deposited within and along collagen fibrils. This common ground of collagens seems to explain the coexistence of ectopic mineralization and fibrosis. In a case of septic myocardial calcification, a wide calcification area on the right atrial wall contains the deposition of fibrin and surrounded by fibrotic tissue.^95^

Cardiac fibroblasts (CFs) possess a degree of native cellular plasticity and have central roles in pathogenic remodelling. During myocardial ischaemia, fibroblasts can replace non‐regenerative myocardium upon injury and be activated into contractile smooth muscle‐like myofibroblasts which create a reparative fibrotic scar to prevent ventricular wall rupture.^96^, ^97^ Cardiac fibroblasts can also adopt endothelial cell–like fates through mesenchymal‐endothelial transition (MEndoT) following acute cardiac injury, and fibroblast‐derived endothelial cells have an important role in promoting the neovascularization of injured heart and cardiac repair. ^98^ This transition can be augmented by p53, a cellular stress‐response gene, which may have a profound effect on reprogramming. ^99^

Recently, Pallai et al reported that cardiac fibroblasts can adopt an osteoblast cell‐like fates, contributing to cardiac calcification after injury.^90^ This study illustrated the potential of cardiac fibroblasts converting to osteoblast‐like cell with an in vitro cell culture system. Culturing adult C57/BL6 CFs with osteogenic differentiation medium (DM) led to the deposition of calcium hydroxyapatite with the expression of canonical osteoblast genes (Runx2, osteocalcin, osterix, bone sialoprotein and osteopontin). In vivo, three murine models of C3H strain (high‐dose steroids, cryo‐injury and ischaemic injury) were created. Murine hearts exhibited calcium hydroxyapatite deposition in injured regions where there was fibrosis. In border region, genetically labelled CFs expressed osteoblast markers and Runx2, the master osteogenic transcription factor. This report suggests that cardiac fibroblasts possess a high degree of plasticity and can adopt an osteogenic phenotype. Notably, some strains of mice like B6 strains failed to exhibit hydroxyapatite deposition post–heart injury. In the meantime, few osteogenic markers were witnessed in labelled CFs of those strains, suggesting that osteogenic fate of CFs is thick with mouse strains.

Researchers also found the expression of ENPP1 was up‐regulated after injury, and inhibition of ENPP1 with small molecules could dramatically decrease ectopic cardiac calcification and preserve cardiac function. This seems to be contrary to the previous opinion that ENPP1 can inhibit ectopic mineralization. Most likely, this is attributed to the enrichment of TNAP in myocardium that can hydrolyse PPi to generate Pi, whereas Pi can be used as constituents of hydroxyapatite.

## 6 THE MAIN SIGNALLING GOVERNING FIBROBLAST‐MEDIATED CALCIFICATION

**FIGURE 1:**
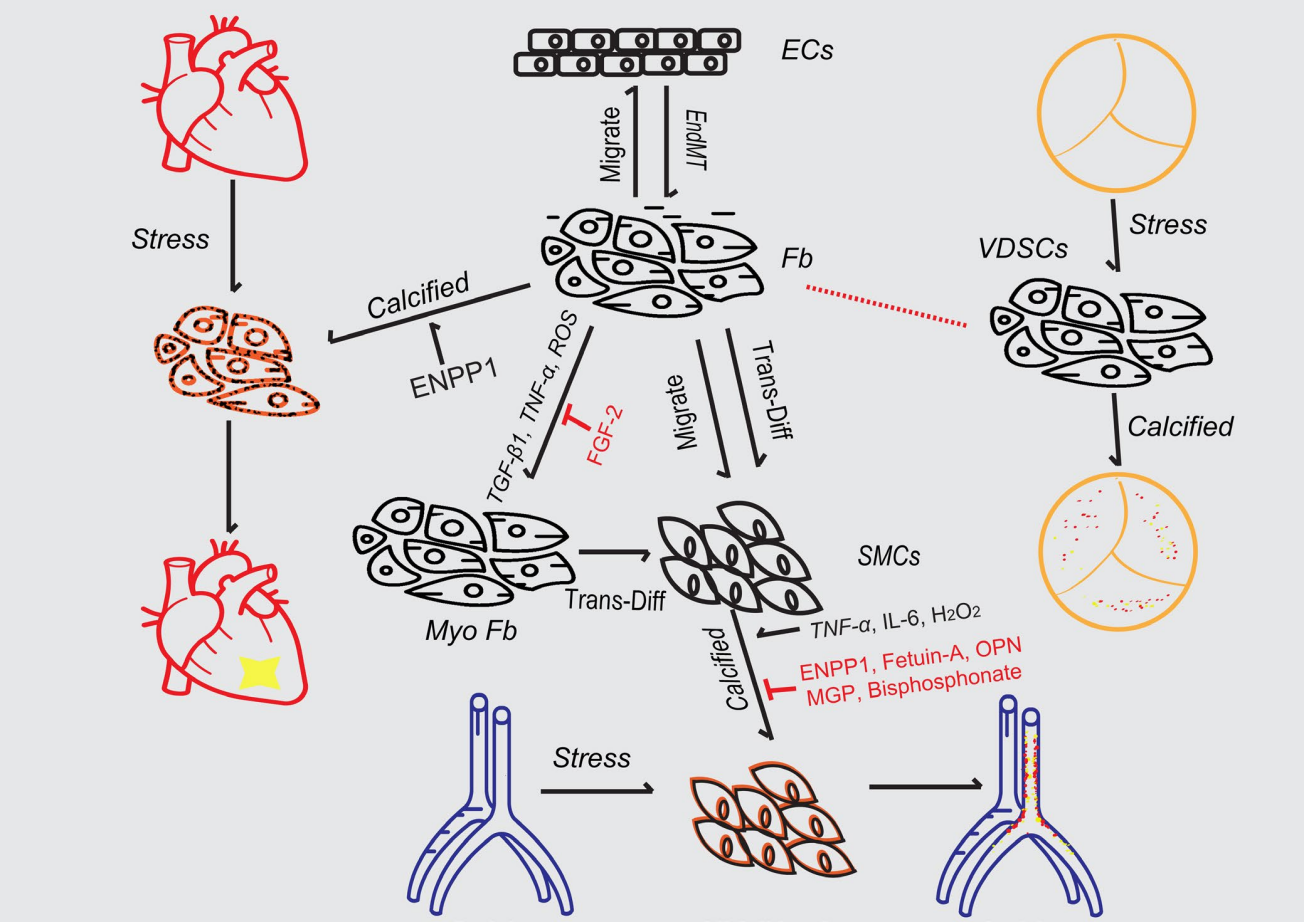
Roles of fibroblasts in cardiac and vascular calcification. Schematic representation of the central role of fibroblasts in regulating calcification via diverse signalling pathways. ECs, endothelial cells; Fb, fibroblasts; MyoFb, myofibroblasts; SMCs, smooth muscle cells; VDSCs, valve‐derived stromal cells; VICs, valve interstitial cells; EndMT, endothelial‐to‐mesenchymal transition; TGF‐β1, transforming growth factor‐beta1; ENPP1, ectonucleotide pyrophosphatase/phosphodiesterase‐1; TNF‐α, tumour necrosis factor‐α; ROS, reactive oxygen species; IL‐6, interleukin‐6; FGF‐2, fibroblast growth factor‐2; H_2_O_2,_ hydrogen peroxide; OPN, osteopontin; MGP, matrix Gla protein

As discussed above, fibroblasts play a central role in tissue calcification under diverse conditions, like ischaemia, pressure overload, shear stress and inflammation. Fibroblasts can trans‐differentiate into myofibroblasts with enhanced abilities of proliferation and migration under the stimulus of numerous growth factors and cytokines, such as ROS, TGF‐β1 and TNF‐α. Vascular smooth muscle cells can switch into calcified phenotype within stimulus. For example, H_2_O_2_ can induce the osteogenic differentiation of VSMCs by increasing the expression of Runx2_,_ a vital osteogenic transcription factor. TNF‐α promotes arterial calcification by activating Msx2‐Wnt signalling, and IL‐6 induces the osteogenic differentiation of VSMCs through STAT3/JMJD2B pathway, whereas fetuin‐A, ENPP1, MGP, osteopontin and pyrophosphate analogues can inhibit vascular calcification by similar mechanisms, including re‐balancing calcium‐phosphate metabolism and inhibiting the formation and propagation of hydroxyapatite crystals. Interestingly, ENPP1, a PPi‐generating ectophosphatase, can strongly inhibit ectopic mineralization. As PPi can directly inhibit the aggregation and growth of hydroxyapatite crystal, it was supposed that ENPP1 should inhibit the calcification of heart after injury. However, PPi can be hydrolysed into Pi by TNAP in myocardium, whereas pi is the main component of hydroxyapatite crystals, which may result in the unexpected promotive roles of ENPP1 in myocardium mineralization. In general, ectopic calcification is a gambling process between pro‐calcification stimuli and anti‐calcification factors. It is emerging to explore the further molecular mechanism which highlights the plasticity of fibroblasts in contributing to ectopic calcification and identify pharmacological targets for therapy (Figure 1).

## 7 CONCLUSION

In recent decades, cardiovascular calcification, including vascular, valvular and myocardial calcification, has attracted dramatically increasing attention. Vascular calcification is characterized as the deposition of hydroxyapatite in intima and media of arterial wall majorly triggered by the osteogenic differentiation of VSMCs. Valve calcification is triggered by valve cells, accompanied by dysfunction of valve leaflets, and mainly affects mitral and aortic valves. Myocardial calcification with hydroxyapatite deposited within the myocardium is often accompanied by poor outcome. All these cardiovascular events share similar risk factors such as hypertension, hyperlipidaemia and diabetes. Intimal calcification is always observed in atherosclerotic lesions, and atherosclerosis shares many commonalities with mitral annular calcification. Some scientists believed that MAC is another form of atherosclerosis. Besides intimal calcification frequently occurs in the coronary arteries, stenosis or obstruction of coronary can lead to impaired heart function or sudden cardiac death.

Fibroblasts with potent plasticity are critical in cardiovascular remodelling. Fibroblasts from different organs exhibit similar morphology and functions, including secreting cytokines and numerous extracellular matrix proteins, as well as displaying potent plasticity. In the progression of atherosclerosis, adventitial fibroblasts trans‐differentiate into myofibroblasts and migrate towards the lumen, contributing to the formation of neointima, and it also displays paracrine effects on VSMSs calcification via a set of cytokine release, including H_2_O_2_, inflammatory factors and VEGF. Inflammatory activity and angiogenesis play a contributing role in VSMSs calcification. Local treatment targeting anti‐inflammatory and anti‐angiogenesis through adventitia may have an inhibitory effect on vascular calcification. In valve calcification, valve interstitial cells undergoing osteogenic differentiation are recognized as the major cellular origin of calcific valve. Recently, scientists discovered that valve‐derived stromal cells, as fibroblast‐like cells, expressing both mesenchymal and osteogenic markers, may play a contributing role in valve calcification. Myocardial calcification, with minerals deposited in the myocardium, is a severe complication of cardiac injury whereas few is known regarding the cellular source of calcification. Pallai et al revealed that cardiac fibroblasts undergoing osteogenic fate critically contribute to the calcification of heart muscle. Inhibition of ENPP1 could dramatically attenuate cardiac calcification.

This review, for the first time, systemically summarizes the emerging roles of fibroblasts in cardiovascular calcification and discusses the potential therapeutics targeting fibroblasts in cardiovascular calcification. It is noteworthy that the lack of specific markers to identify fibroblasts makes it difficult to explore the precise features of fibroblasts. The markers identifying fibroblasts, such as fibroblast‐specific protein‐1 (FSP‐1), collagen 1a1 and transcription factor 21 (TCF21), are not uniformly specific. They are also expressed on VSMCs, ECs and immune cells.^100^ Periostin, a newly recognized marker of myofibroblasts, is expressed in adult heart tissues only after injury. Further study identified that periostin^+^ myofibroblasts in heart are originated from TCF21^+^ tissue–resident fibroblasts.^97^ Since the complex of fibroblast plasticity, more specific markers need to be explored, a combination of two or more fibrotic markers is recommended to label fibroblasts in tissues. Future studies will further our knowledge of fibroblasts in different diseases.

## CONFLICTS OF INTEREST

The authors declared no conflict of interest.

## AUTHOR CONTRIBUTION

**Wudi Li:** Writing‐review & editing (lead). **Sheng‐an Su:** Writing‐review & editing (lead). **Jian Chen:** Writing‐review & editing (supporting). **Hong Ma:** Supervision (equal); Writing‐review & editing (supporting). **Meixiang Xiang:** Funding acquisition (lead); Supervision (equal).

## References

[1] Benjamin EJ , Muntner P , Alonso A , et al. Heart disease and stroke statistics‐2019 update: a report from the American Heart Association. Circulation. 2019;139:e56‐e528. 30700139 10.1161/CIR.0000000000000659

[2] Roth GA , Johnson C , Abajobir A , et al. Global, regional, and national burden of cardiovascular diseases for 10 causes, 1990 to 2015. J Am Coll Cardiol. 2017;70:1‐25. 28527533 10.1016/j.jacc.2017.04.052 PMC5491406

[3] Rogers MA , Aikawa E . Cardiovascular calcification: artificial intelligence and big data accelerate mechanistic discovery. Nat Rev Cardiol. 2019;16:261‐274. 30531869 10.1038/s41569-018-0123-8

[4] Demer LL , Tintut Y . Inflammatory, metabolic, and genetic mechanisms of vascular calcification. Arterioscler Thromb Vasc Biol. 2014;34:715‐723. 24665125 10.1161/ATVBAHA.113.302070 PMC3975044

[5] Hortells L , Sur S , St HC . Cell phenotype transitions in cardiovascular calcification. Front Cardiovasc Med. 2018;5:27. 29632866 10.3389/fcvm.2018.00027 PMC5879740

[6] Suyama T , Hatta M , Hata S , et al. Differentiation of rat dermal mesenchymal cells and calcification in three‐dimensional cultures. Tissue Eng Regen Med. 2016;13:527‐537. 30603433 10.1007/s13770-016-9124-z PMC6170832

[7] Lev M . Anatomic basis for atrioventricular block. Am J Med. 1964;37:742‐748. 14237429 10.1016/0002-9343(64)90022-1

[8] Fathi I , Sakr M . Review of tumoral calcinosis: a rare clinico‐pathological entity. World J Clin Cases. 2014;2:409‐414. 25232542 10.12998/wjcc.v2.i9.409 PMC4163761

[9] Sha Y , Hong K , Liew MKM , et al. Juxta‐articular tumoral calcinosis associated with the temporomandibular joint: a case report and concise review. BMC Oral Health. 2019;19:138. 31288794 10.1186/s12903-019-0816-3 PMC6617841

[10] Fleisch H , Russell RG , Straumann F . Effect of pyrophosphate on hydroxyapatite and its implications in calcium homeostasis. Nature. 1966;212:901‐903. 4306793 10.1038/212901a0

[11] Fleisch H , Bisaz S . Mechanism of calcification: inhibitory role of pyrophosphate. Nature. 1962;195:911. 10.1038/195911a0 13893487

[12] Luo G , Ducy P , McKee MD , et al. Spontaneous calcification of arteries and cartilage in mice lacking matrix GLA protein. Nature. 1997;386:78‐81. 9052783 10.1038/386078a0

[13] Speer MY , McKee MD , Guldberg RE , et al. Inactivation of the osteopontin gene enhances vascular calcification of matrix Gla protein‐deficient mice: evidence for osteopontin as an inducible inhibitor of vascular calcification in vivo. J Exp Med. 2002;196:1047‐1055. 12391016 10.1084/jem.20020911 PMC2194039

[14] Rutsch F , Ruf N , Vaingankar S , et al. Mutations in ENPP1 are associated with 'idiopathic' infantile arterial calcification. Nat Genet. 2003;34:379‐381. 12881724 10.1038/ng1221

[15] Price PA , Roublick AM , Williamson MK . Artery calcification in uremic rats is increased by a low protein diet and prevented by treatment with ibandronate. Kidney Int. 2006;70:1577‐1583. 16955099 10.1038/sj.ki.5001841

[16] Heiss A , DuChesne A , Denecke B , et al. Structural basis of calcification inhibition by alpha 2‐HS glycoprotein/fetuin‐A. Formation of colloidal calciprotein particles. J Biol Chem. 2003;278:13333‐13341. 12556469 10.1074/jbc.M210868200

[17] Schafer C , Heiss A , Schwarz A , et al. The serum protein alpha 2‐Heremans‐Schmid glycoprotein/fetuin‐A is a systemically acting inhibitor of ectopic calcification. J Clin Invest. 2003;112:357‐366. 12897203 10.1172/JCI17202 PMC166290

[18] Wada T , McKee MD , Steitz S , et al. Calcification of vascular smooth muscle cell cultures: inhibition by osteopontin. Circ Res. 1999;84:166‐178. 9933248 10.1161/01.res.84.2.166

[19] Yahagi K , Kolodgie FD , Lutter C , et al. Pathology of Human Coronary and Carotid Artery Atherosclerosis and Vascular Calcification in Diabetes Mellitus. Arterioscler Thromb Vasc Biol. 2017;37:191‐204. 27908890 10.1161/ATVBAHA.116.306256 PMC5269516

[20] Sarwar N , Gao P , Seshasai SR , et al. Diabetes mellitus, fasting blood glucose concentration, and risk of vascular disease: a collaborative meta‐analysis of 102 prospective studies. Lancet. 2010;375:2215‐2222. 20609967 10.1016/S0140-6736(10)60484-9 PMC2904878

[21] London GM , Guerin AP , Marchais SJ , et al. Arterial media calcification in end‐stage renal disease: impact on all‐cause and cardiovascular mortality. Nephrol Dial Transplant. 2003;18:1731‐1740. 12937218 10.1093/ndt/gfg414

[22] Hu Y , Zhang Z , Torsney E , et al. Abundant progenitor cells in the adventitia contribute to atherosclerosis of vein grafts in ApoE‐deficient mice. J Clin Invest. 2004;113:1258‐1265. 15124016 10.1172/JCI19628 PMC398426

[23] Hu Y , Xu Q . Adventitial biology: differentiation and function. Arterioscler Thromb Vasc Biol. 2011;31:1523‐1529. 21677295 10.1161/ATVBAHA.110.221176

[24] Ambale‐Venkatesh B , Yang X , Wu CO , et al. Cardiovascular Event Prediction by Machine Learning: The Multi‐Ethnic Study of Atherosclerosis. Circ Res. 2017;121:1092‐1101. 28794054 10.1161/CIRCRESAHA.117.311312 PMC5640485

[25] Polonsky TS , Blumenthal RS , Greenland P . Coronary artery calcium score. JAMA. 2014;312:837‐838. 25157727 10.1001/jama.2014.1948

[26] Foks AC , Bot I . Preface: Pathology and Pharmacology of Atherosclerosis. Eur J Pharmacol. 2017;816:1‐2. 29108620 10.1016/j.ejphar.2017.10.052

[27] Lanzer P , Boehm M , Sorribas V , et al. Medial vascular calcification revisited: review and perspectives. Eur Heart J. 2014;35:1515‐1525. 24740885 10.1093/eurheartj/ehu163 PMC4072893

[28] Steitz SA , Speer MY , Curinga G , et al. Smooth muscle cell phenotypic transition associated with calcification: upregulation of Cbfa1 and downregulation of smooth muscle lineage markers. Circ Res. 2001;89:1147‐1154. 11739279 10.1161/hh2401.101070

[29] Durham AL , Speer MY , Scatena M , et al. Role of smooth muscle cells in vascular calcification: implications in atherosclerosis and arterial stiffness. Cardiovasc Res. 2018;114:590‐600. 29514202 10.1093/cvr/cvy010 PMC5852633

[30] Oshima J , Sidorova JM , Monnat RJ Jr . Werner syndrome: Clinical features, pathogenesis and potential therapeutic interventions. Ageing Res Rev. 2017;33:105‐114. 26993153 10.1016/j.arr.2016.03.002 PMC5025328

[31] Boraldi F , Bartolomeo A , Li Q , et al. Changes in dermal fibroblasts from Abcc6(‐/‐) mice are present before and after the onset of ectopic tissue mineralization. J Invest Dermatol. 2014;134:1855‐1861. 24670382 10.1038/jid.2014.88 PMC4057957

[32] Okubo Y , Masuyama R , Iwanaga A , et al. Calcification in dermal fibroblasts from a patient with GGCX syndrome accompanied by upregulation of osteogenic molecules. PLoS One. 2017;12:e0177375. 28494010 10.1371/journal.pone.0177375 PMC5426700

[33] Ronchetti I , Boraldi F , Annovi G , et al. Fibroblast involvement in soft connective tissue calcification. Front Genet. 2013;4:22. 23467434 10.3389/fgene.2013.00022 PMC3588566

[34] Dabisch‐Ruthe M , Kuzaj P , Götting C , et al. Pyrophosphates as a major inhibitor of matrix calcification in *Pseudoxanthoma elasticum* . J Dermatol Sci. 2014;75:109‐120. 24907773 10.1016/j.jdermsci.2014.04.015

[35] Simionescu A , Simionescu DT , Vyavahare NR . Osteogenic responses in fibroblasts activated by elastin degradation products and transforming growth factor‐beta1: role of myofibroblasts in vascular calcification. Am J Pathol. 2007;171:116‐123. 17591959 10.2353/ajpath.2007.060930 PMC1941602

[36] Boraldi F , Annovi G , Bartolomeo A , et al. Fibroblasts from patients affected by Pseudoxanthoma elasticum exhibit an altered PPi metabolism and are more responsive to pro‐calcifying stimuli. J Dermatol Sci. 2014;74:72‐80. 24461675 10.1016/j.jdermsci.2013.12.008

[37] Honjo S , Yokote K , Fujimoto M , et al. Clinical outcome and mechanism of soft tissue calcification in Werner syndrome. Rejuvenation Res. 2008;11:809‐819. 18729813 10.1089/rej.2007.0649

[38] Kalantari F , Miao D , Emadali A , et al. Cellular and molecular mechanisms of abnormal calcification following ischemia‐reperfusion injury in human liver transplantation. Modern Pathol. 2007;20:357‐366. 10.1038/modpathol.3800747 17334330

[39] Talmon GA , Wisecarver JL . Hepatocellular calcification in severe ischemia‐reperfusion injury in a liver allograft. Ultrastruct Pathol. 2010;34:362‐365. 21070168 10.3109/01913123.2010.506254

[40] Tzimas GN , Afshar M , Chevet E , et al. Graft calcifications and dysfunction following liver transplantation. BMC Surg. 2004;4:9. 15347427 10.1186/1471-2482-4-9 PMC517503

[41] Yu F , Cui Y , Zhou X , et al. Osteogenic differentiation of human ligament fibroblasts induced by conditioned medium of osteoclast‐like cells. Biosci Trends. 2011;5:46‐51. 21572246 10.5582/bst.2011.v5.2.46

[42] Li DH , He CR , Liu FP , et al. Annexin A2, up‐regulated by IL‐6, promotes the ossification of ligament fibroblasts from ankylosing spondylitis patients. Biomed Pharmacother. 2016;84:674‐679. 27697640 10.1016/j.biopha.2016.09.091

[43] Chen D , Liu Y , Yang H , et al. Connexin 43 promotes ossification of the posterior longitudinal ligament through activation of the ERK1/2 and p38 MAPK pathways. Cell Tissue Res. 2016;363:765‐773. 26334722 10.1007/s00441-015-2277-6

[44] Kelly‐Arnold A , Maldonado N , Laudier D , et al. Revised microcalcification hypothesis for fibrous cap rupture in human coronary arteries. Proc Natl Acad Sci USA. 2013;110:10741‐10746. 23733926 10.1073/pnas.1308814110 PMC3696743

[45] Vengrenyuk Y , Carlier S , Xanthos S , et al. A hypothesis for vulnerable plaque rupture due to stress‐induced debonding around cellular microcalcifications in thin fibrous caps. Proc Natl Acad Sci USA. 2006;103:14678‐14683. 17003118 10.1073/pnas.0606310103 PMC1595411

[46] Maldonado N , Kelly‐Arnold A , Vengrenyuk Y , et al. A mechanistic analysis of the role of microcalcifications in atherosclerotic plaque stability: potential implications for plaque rupture. Am J Physiol Heart Circ Physiol. 2012;303:H619‐628. 22777419 10.1152/ajpheart.00036.2012 PMC3468470

[47] Cheng GC , Loree HM , Kamm RD , et al. Distribution of circumferential stress in ruptured and stable atherosclerotic lesions. A structural analysis with histopathological correlation. Circulation. 1993;87:1179‐1187. 8462145 10.1161/01.cir.87.4.1179

[48] Li CJ , Sun HW , Zhu FL , et al. Local adiponectin treatment reduces atherosclerotic plaque size in rabbits. J Endocrinol. 2007;193:137‐145. 17400811 10.1677/JOE-06-0173

[49] Yuen CY , Wong SL , Lau CW , et al. From skeleton to cytoskeleton: osteocalcin transforms vascular fibroblasts to myofibroblasts via angiotensin II and Toll‐like receptor 4. Circ Res. 2012;111:e55‐66. 22679141 10.1161/CIRCRESAHA.112.271361

[50] Gao PJ , Li Y , Sun AJ , et al. Differentiation of vascular myofibroblasts induced by transforming growth factor‐beta1 requires the involvement of protein kinase Calpha. J Mol Cell Cardiol. 2003;35:1105‐1112. 12967633 10.1016/s0022-2828(03)00207-4

[51] He Y , Xiao Y , Yang X , et al. SIRT6 inhibits TNF‐alpha‐induced inflammation of vascular adventitial fibroblasts through ROS and Akt signaling pathway. Exp Cell Res. 2017;357:88‐97. 28477980 10.1016/j.yexcr.2017.05.001

[52] Liguori T , Liguori GR , Moreira L , et al. Fibroblast growth factor‐2, but not the adipose tissue‐derived stromal cells secretome, inhibits TGF‐beta1‐induced differentiation of human cardiac fibroblasts into myofibroblasts. Sci Rep. 2018;8:16633. 30413733 10.1038/s41598-018-34747-3 PMC6226511

[53] Li G , Chen SJ , Oparil S , et al. Direct in vivo evidence demonstrating neointimal migration of adventitial fibroblasts after balloon injury of rat carotid arteries. Circulation. 2000;101:1362‐1365. 10736277 10.1161/01.cir.101.12.1362

[54] Karamariti E , Zhai C , Yu B , et al. DKK3 (Dickkopf 3) alters atherosclerotic plaque phenotype involving vascular progenitor and fibroblast differentiation into smooth muscle cells. Arterioscler Thromb Vasc Biol. 2018;38:425‐437. 29284609 10.1161/ATVBAHA.117.310079

[55] Evrard SM , Lecce L , Michelis KC , et al. Endothelial to mesenchymal transition is common in atherosclerotic lesions and is associated with plaque instability. Nat Commun. 2016;7:11853. 27340017 10.1038/ncomms11853 PMC4931033

[56] Stenmark KR , Yeager ME , El KK , et al. The adventitia: essential regulator of vascular wall structure and function. Annu Rev Physiol. 2013;75:23‐47. 23216413 10.1146/annurev-physiol-030212-183802 PMC3762248

[57] Dourron HM , Jacobson GM , Park JL , et al. Perivascular gene transfer of NADPH oxidase inhibitor suppresses angioplasty‐induced neointimal proliferation of rat carotid artery. Am J Physiol Heart Circ Physiol. 2005;288:H946‐953. 15388496 10.1152/ajpheart.00413.2004

[58] Li XD , Hong MN , Chen J , et al. Adventitial fibroblast‐derived vascular endothelial growth factor promotes vasa vasorum‐associated neointima formation and macrophage recruitment. Cardiovasc Res. 2020;116:708‐720. 31241138 10.1093/cvr/cvz159

[59] Langheinrich AC , Michniewicz A , Sedding DG , et al. Correlation of vasa vasorum neovascularization and plaque progression in aortas of apolipoprotein E(‐/‐)/low‐density lipoprotein(‐/‐) double knockout mice. Arterioscler Thromb Vasc Biol. 2006;26:347‐352. 16293797 10.1161/01.ATV.0000196565.38679.6d

[60] Tziakas DN , Chalikias G , Pavlaki M , et al. Lysed erythrocyte membranes promote vascular calcification. Circulation. 2019;139:2032‐2048. 30717607 10.1161/CIRCULATIONAHA.118.037166

[61] Foresta C , Strapazzon G , De Toni L , et al. Platelets express and release osteocalcin and co‐localize in human calcified atherosclerotic plaques. J Thromb Haemost. 2013;11:357‐365. 23206207 10.1111/jth.12088

[62] Idelevich A , Rais Y , Monsonego‐Ornan E . Bone Gla protein increases HIF‐1alpha‐dependent glucose metabolism and induces cartilage and vascular calcification. Arterioscler Thromb Vasc Biol. 2011;31:e55‐71. 21757657 10.1161/ATVBAHA.111.230904

[63] Bouchareb R , Boulanger MC , Tastet L , et al. Activated platelets promote an osteogenic programme and the progression of calcific aortic valve stenosis. Eur Heart J. 2019;40:1362‐1373. 30395215 10.1093/eurheartj/ehy696 PMC6492053

[64] Moulton KS , Vakili K , Zurakowski D , et al. Inhibition of plaque neovascularization reduces macrophage accumulation and progression of advanced atherosclerosis. Proc Natl Acad Sci USA. 2003;100:4736‐4741. 12682294 10.1073/pnas.0730843100 PMC153625

[65] Haurani MJ , Pagano PJ . Adventitial fibroblast reactive oxygen species as autacrine and paracrine mediators of remodeling: bellwether for vascular disease? Cardiovasc Res. 2007;75:679‐689. 17689510 10.1016/j.cardiores.2007.06.016

[66] Stenmark KR , Davie N , Frid M , et al. Role of the adventitia in pulmonary vascular remodeling. Physiology (Bethesda). 2006;21:134‐145. 16565479 10.1152/physiol.00053.2005

[67] Byon CH , Javed A , Dai Q , et al. Oxidative stress induces vascular calcification through modulation of the osteogenic transcription factor Runx2 by AKT signaling. J Biol Chem. 2008;283:15319‐15327. 18378684 10.1074/jbc.M800021200 PMC2397455

[68] Zhang L , Zalewski A , Liu Y , et al. Diabetes‐induced oxidative stress and low‐grade inflammation in porcine coronary arteries. Circulation. 2003;108:472‐478. 12860917 10.1161/01.CIR.0000080378.96063.23

[69] Al‐Aly Z , Shao JS , Lai CF , et al. Aortic Msx2‐Wnt calcification cascade is regulated by TNF‐alpha‐dependent signals in diabetic Ldlr‐/‐ mice. Arterioscler Thromb Vasc Biol. 2007;27:2589‐2596. 17932314 10.1161/ATVBAHA.107.153668

[70] Kurozumi A , Nakano K , Yamagata K , et al. IL‐6 and sIL‐6R induces STAT3‐dependent differentiation of human VSMCs into osteoblast‐like cells through JMJD2B‐mediated histone demethylation of RUNX2. Bone. 2019;124:53‐61. 30981888 10.1016/j.bone.2019.04.006

[71] Goldbarg SH , Elmariah S , Miller MA , et al. Insights into degenerative aortic valve disease. J Am Coll Cardiol. 2007;50:1205‐1213. 17888836 10.1016/j.jacc.2007.06.024

[72] Abramowitz Y , Jilaihawi H , Chakravarty T , et al. Mitral Annulus Calcification. J Am Coll Cardiol. 2015;66:1934‐1941. 26493666 10.1016/j.jacc.2015.08.872

[73] Ben ZS , Freeman J , Jajoo A , et al. Patient‐specific quantitation of mitral valve strain by computer analysis of three‐dimensional echocardiography: a pilot study. Circ Cardiovasc Imaging. 2016;9(1):e003254. 26712158 10.1161/CIRCIMAGING.115.003254

[74] Bortnick AE , Bartz TM , Ix JH , et al. Association of inflammatory, lipid and mineral markers with cardiac calcification in older adults. Heart. 2016;102:1826‐1834. 27411840 10.1136/heartjnl-2016-309404 PMC5235996

[75] Carrizzo A , Izzo C , Oliveti M , et al. The main determinants of diabetes mellitus vascular complications: endothelial dysfunction and platelet hyperaggregation. Int J Mol Sci. 2018;19:2968. 10.3390/ijms19102968 PMC6212935 30274207

[76] Adler Y , Herz I , Vaturi M , et al. Mitral annular calcium detected by transthoracic echocardiography is a marker for high prevalence and severity of coronary artery disease in patients undergoing coronary angiography. Am J Cardiol. 1998;82:1183‐1186. 9832091 10.1016/s0002-9149(98)00596-7

[77] Roberts WC . The senile cardiac calcification syndrome. Am J Cardiol. 1986;58:572‐574. 3751927 10.1016/0002-9149(86)90045-7

[78] O'Neal WT , Efird JT , Nazarian S , et al. Mitral annular calcification and incident atrial fibrillation in the Multi‐Ethnic Study of Atherosclerosis. Europace. 2015;17:358‐363. 25341740 10.1093/europace/euu265 PMC4415068

[79] Beaudoin J , Dal‐Bianco JP , Aikawa E , et al. Mitral leaflet changes following myocardial infarction: clinical evidence for maladaptive valvular remodeling. Circ Cardiovasc Imaging. 2017;10(11):e006512. 29042413 10.1161/CIRCIMAGING.117.006512 PMC5683411

[80] Fox CS , Vasan RS , Parise H , et al. Mitral annular calcification predicts cardiovascular morbidity and mortality: the Framingham Heart Study. Circulation. 2003;107:1492‐1496. 12654605 10.1161/01.cir.0000058168.26163.bc

[81] Kohsaka S , Jin Z , Rundek T , et al. Impact of mitral annular calcification on cardiovascular events in a multiethnic community: the Northern Manhattan Study. JACC Cardiovasc Imaging. 2008;1:617‐623. 19356491 10.1016/j.jcmg.2008.07.006 PMC2847358

[82] Otto CM , Kuusisto J , Reichenbach DD , et al. Characterization of the early lesion of 'degenerative' valvular aortic stenosis. Histological and immunohistochemical studies. Circulation. 1994;90:844‐853. 7519131 10.1161/01.cir.90.2.844

[83] Rutkovskiy A , Malashicheva A , Sullivan G , et al. Valve interstitial cells: the key to understanding the pathophysiology of heart valve calcification. J Am Heart Assoc. 2017;6(9):e006339. 28912209 10.1161/JAHA.117.006339 PMC5634284

[84] Huang Y , Xu K , Zhou T , et al. Comparison of rapidly proliferating, multipotent aortic valve‐derived stromal cells and valve interstitial cells in the human aortic valve. Stem Cells Int. 2019;2019:7671638. 31582988 10.1155/2019/7671638 PMC6754971

[85] Wylie‐Sears J , Aikawa E , Levine RA , et al. Mitral valve endothelial cells with osteogenic differentiation potential. Arterioscler Thromb Vasc Biol. 2011;31:598‐607. 21164078 10.1161/ATVBAHA.110.216184 PMC3210435

[86] Hjortnaes J , Shapero K , Goettsch C , et al. Valvular interstitial cells suppress calcification of valvular endothelial cells. Atherosclerosis. 2015;242:251‐260. 26232165 10.1016/j.atherosclerosis.2015.07.008 PMC4546848

[87] Na JY . A heart of stone: an autopsy case of massive myocardial calcification. Forensic Sci Med Pathol. 2018;14:102‐105. 29197949 10.1007/s12024-017-9936-8

[88] Austin CO , Kramer D , Canabal J , et al. A heart of stone: a case of acute development of cardiac calcification and hemodynamic collapse. J Cardiovasc Comput Tomogr. 2013;7:66‐68. 23394819 10.1016/j.jcct.2012.07.004

[89] Stallion A , Rafferty JF , Warner BW , et al. Myocardial calcification: a predictor of poor outcome for myocarditis treated with extracorporeal life support. J Pediatr Surg. 1994;29:492‐494. 8014800 10.1016/0022-3468(94)90074-4

[90] Pillai I , Li S , Romay M , et al. Cardiac fibroblasts adopt osteogenic fates and can be targeted to attenuate pathological heart calcification. Cell Stem Cell. 2017;20(218–232):e215. 10.1016/j.stem.2016.10.005 PMC5291784 27867037

[91] Pinto AR , Ilinykh A , Ivey MJ , et al. Revisiting cardiac cellular composition. Circ Res. 2016;118:400‐409. 26635390 10.1161/CIRCRESAHA.115.307778 PMC4744092

[92] Moore‐Morris T , Guimaraes‐Camboa N , Yutzey KE , et al. Cardiac fibroblasts: from development to heart failure. J Mol Med (Berl). 2015;93:823‐830. 26169532 10.1007/s00109-015-1314-y PMC4512919

[93] Travers JG , Kamal FA , Robbins J , et al. Cardiac fibrosis: the fibroblast awakens. Circ Res. 2016;118:1021‐1040. 26987915 10.1161/CIRCRESAHA.115.306565 PMC4800485

[94] Ma ZG , Yuan YP , Wu HM , et al. Cardiac fibrosis: new insights into the pathogenesis. Int J Biol Sci. 2018;14:1645‐1657. 30416379 10.7150/ijbs.28103 PMC6216032

[95] Maiese A , Manetti F , La Russa R , et al. Septic myocardial calcification: a case report. J Forensic Leg Med. 2019;65:45‐47. 31100653 10.1016/j.jflm.2019.05.004

[96] van den Borne SW , Diez J , Blankesteijn WM , et al. Myocardial remodeling after infarction: the role of myofibroblasts. Nat Rev Cardiol. 2010;7:30‐37. 19949426 10.1038/nrcardio.2009.199

[97] Kanisicak O , Khalil H , Ivey MJ , et al. Genetic lineage tracing defines myofibroblast origin and function in the injured heart. Nat Commun. 2016;7:12260. 27447449 10.1038/ncomms12260 PMC5512625

[98] Ubil E , Duan J , Pillai IC , et al. Mesenchymal‐endothelial transition contributes to cardiac neovascularization. Nature. 2014;514:585‐590. 25317562 10.1038/nature13839 PMC4214889

[99] Hong H , Takahashi K , Ichisaka T , et al. Suppression of induced pluripotent stem cell generation by the p53–p21 pathway. Nature. 2009;460:1132‐1135. 19668191 10.1038/nature08235 PMC2917235

[100] Kong P , Christia P , Saxena A , et al. Lack of specificity of fibroblast‐specific protein 1 in cardiac remodeling and fibrosis. Am J Physiol Heart Circ Physiol. 2013;305:H1363‐1372. 23997102 10.1152/ajpheart.00395.2013 PMC3840245

